# Effects of Hypercarbia on Lower Extremity Primary Total Joint Replacement Infections

**DOI:** 10.7759/cureus.26069

**Published:** 2022-06-18

**Authors:** Teigen Goodeill, Justin Than, Olivia Pipitone, Jason Lin, Nicholas Tedesco

**Affiliations:** 1 Orthopedic Surgery, Good Samaritan Regional Medical Center, Corvallis, USA; 2 Graduate Medical Education - Biostatistician, Good Samaritan Regional Medical Center, Corvallis, USA

**Keywords:** prosthetic joint infection, surgical site injection, hypercarbia, total joint replacement, total joint arthroplasty, peri-prosthetic joint infection

## Abstract

Introduction: Prosthetic joint infection (PJI) is a serious complication after total joint replacement (TJR). Adequate wound oxygenation is critical for wound healing and infection prevention. As carbon dioxide (CO_2_) is exchanged for oxygen (O_2_) in the lungs, serum bicarbonate (HCO_3_^-^) may be used as a marker for predicting relative serum O_2_ levels, and therefore, healing potential. No currently published literature explores the relationship between serum bicarbonate levels and PJI in TJR patients.

Methods: We performed this retrospective review of lower extremity TJR patients to determine whether the risk of PJI and wound complications within one year was correlated with hypercarbia, which was defined as a preoperative serum bicarbonate level >30 mEq/L.

Results: Out of 1,690 TJR procedures, 1.6% (N=27) had a PJI or superficial wound infection within one year postoperatively. The average preoperative serum bicarbonate was 26.9 (SD 2.6) among patients without PJI and 27.2 (SD 2.1) among patients with PJI (p=0.46). Hypercarbia was present in 9.2% of non-PJI patients and in 7.4% of PJI patients. The relative risk of PJI and wound complications did not differ for patients with vs without hypercarbia (RR = 0.79, 95% CI = 0.19-3.31, p=0.75).

Conclusion: The results of this study provide preliminary evidence that preoperative hypercarbia may not be correlated with an increase in the risk of PJI or wound complications. However, due to the rarity of both PJI and hypercarbia, a larger patient population is needed to ensure adequate power to detect clinically meaningful effect sizes.

## Introduction

The incidence of total joint replacement (TJR) in the United States continues to rise, making this one of the most common elective orthopedic procedures [1]. A periprosthetic joint infection (PJI) is one of the most serious complications which may occur after TJR, and many steps are taken in order to avoid infection, including preoperative risk mitigation based on identifiable and modifiable risk factors determined from patient histories, comorbidities, and laboratory values.

A measurement that has not been studied as a marker for predicting the risk of infection after TJR is preoperative serum bicarbonate levels. Serum bicarbonate measures the total amount of carbon dioxide in the blood. As blood brings bicarbonate to the lungs, it is exchanged for oxygen (O_2_) and exhaled as carbon dioxide (CO_2_) [2]. Although not the sole reason for an elevation in serum bicarbonate levels, hypoxia is often accompanied by hypercarbia [3]. Diminished oxygen availability causes a decrease in fibroblast proliferation, production of collagen, angiogenesis, re-epithelialization, and the activity of leukocytes, all of which may inhibit healing potential [4]. Additionally, multiple animal studies primarily looking at lung tissue have shown that hypercarbia represses macrophage and immune function activity [5]. Therefore, we hypothesize that higher levels of serum bicarbonate may reflect poor overall oxygenation, and therefore result in higher rates of PJI and wound complications in TJR patients with hypercarbia.

We performed a retrospective review of lower extremity TJR patients to determine whether the risk of PJI and wound complications within one year was correlated with hypercarbia, which was defined as preoperative serum bicarbonate levels >30 mEq/L [2].

## Materials and methods

This is a retrospective cohort study of all lower extremity primary TJR performed at two teaching hospitals in Oregon within the same hospital system between March 2013 and December 2018. The study was reviewed and approved by the Samaritan Health Services Regional Institutional Review Board (IRB19-072). Electronic medical record data was used to identify patients who underwent primary lower extremity joint replacement and had at least one preoperative serum bicarbonate level recorded within 30 days prior to surgery. If the patient had more than one bicarbonate level recorded in that timeframe, the level collected closest to the time of surgery was used. Patients were excluded if they did not have at least one year of follow-up data post-operatively, or if their original surgery was a revision lower extremity TJR, unicompartmental knee arthroplasty, or simultaneous bilateral lower extremity TJR. If a patient had more than one TJR within this timeframe, their first recorded surgery was included.

All patients with an infection diagnosis or a revision surgery within one year after surgery were individually reviewed to confirm the presence of PJI and/or wound complications. Wound healing complications included superficial infection, deep infection, and dehiscence, and were based on definitions established by the United States Center for Disease Control [6].

Patient demographics including age, sex, body mass index (BMI), glycated hemoglobin (HbA1c) levels, smoking status, and data on the procedure site were recorded and analyzed for potential impact on PJI or wound complications. HbA1c samples collected within 90 days before surgery was categorized as greater than seven vs. less than or equal to seven. Each patient’s most recent BMI measurement recorded within 30 days prior to surgery was included. For smoking status, the most recent measurement recorded within six weeks prior to surgery was used.

All analyses were performed in R version 3.6.1 (R Foundation for Statistical Computing, Vienna, Austria). Statistical analysis was performed to compare demographics and bicarbonate levels for patients who had PJI or wound complications with those who did not. Two-sample T-tests were used for continuous variables and Pearson’s Chi-squared tests were used for categorical variables. Fisher’s exact test was employed when the frequency was less than five in any category. Relative risk was calculated to determine whether patients with hypercarbia had a higher risk of PJI or wound complications compared to patients without hypercarbia. In studies such as this, without any previous research to help establish a clinically important difference, an a priori power analysis is inconsequential. Post-hoc power analyses were performed to determine the sample sizes that would be needed in order to have adequate power (80%) at an alpha of 0.05 to detect a statistically significant difference at our observed effect size, and in order to detect a statistically significant difference at effect sizes that may be considered clinically meaningful. 

## Results

A total of 3,315 lower extremity TJR surgeries were performed between March 2013 and December 2018. Based on previously defined criteria 646 surgeries were excluded. An additional 979 patients were excluded because they did not have preoperative serum bicarbonate levels recorded within 30 days before surgery. This left a final study sample of 1,690 patients. Of these, 1.6% (N=27) had a PJI or superficial wound infection within one year postoperatively.

Table 1 shows demographics and serum bicarbonate levels for patients with vs without PJI or wound complications. Most patients underwent total knee replacement and the distribution of procedure sites did not significantly differ across patients with vs without PJI (p=.12). Although patient demographics did not significantly differ across patients with vs without PJI (all p>0.05), the PJI cohort had a higher proportion of males (52% vs 39%), patients with HbA1c over 7 (33% vs 12%), and smokers (15% vs 8%). The average preoperative serum bicarbonate was 26.9 (SD 2.6) among patients without a PJI or wound complication, and 27.2 (SD 2.1) among patients who developed an infection (p=0.46). Out of the total, 9.2% of patients without PJI or wound complications had serum bicarbonate levels >30 mEq/L and 7.4% of patients with an infection had elevated serum bicarbonate levels >30 mEq/L (p>0.99). The relative risk of PJI and wound complications did not differ for patients with vs without hypercarbia (RR = 0.79, 95% CI = 0.19-3.31, p=0.75).

**Table 1:** TJR patients with and without PJI or wound complications within one year of surgery ^1^ p-values are from two-sample t-tests for continuous variables and from Pearson’s Chi-squared tests for categorical variables, or Fisher’s Exact tests when any cell expected frequency was less than 5 ^2^ HbA1c lab results were only available for 450 patients in the no PJI or wound complication group, and for 9 patients in the PJI or wound complication group ^3 ^Hypercarbia is defined as a serum bicarbonate level >30 mEq/L TJR: Total joint replacements, PJI: Prosthetic joint infection, SD: Standard deviation, BMI: Body mass index, HbA1c: Glycated hemoglobin, mEq/L: milliequivalents per liter

	No PJI or wound complication (n=1,663)	PJI or wound complication (n=27)	p-value^1^
Age, mean (SD)	68.4 (10.0)	67.5 (11.0)	0.67
Male, % (n)	38.7% (643)	51.9% (14)	0.23
BMI, mean (SD)	32.2 (6.5)	31.4 (6.6)	0.52
HbA1c >7^2^, % (n)	12.0% (54/450)	33.3% (3/9)	0.09
Smoker, % (n)	7.9% (132)	14.8% (4)	0.27
Serum Bicarbonate Level, mEq/L, mean (SD)	26.9 (2.6)	27.2 (2.0)	0.46
Hypercarbia^3^, % (n)	9.2% (153)	7.4% (2)	>0.99
Procedure Site			0.12
Total Knee Replacement, % (n)	63.3% (1053)	74.1% (20)	
Total Hip Replacement, % (n)	35.5% (591)	22.2% (6)	
Total Ankle Replacement, % (n)	1.1% (19)	3.7% (1)	

For our estimate of relative risk, a post-hoc power analysis showed that, at our observed effect size (1.3% of patients with hypercarbia developing PJI and 1.6% of patients without hypercarbia developing PJI), and assuming our observed ratio of one patient with hypercarbia for every ten patients without hypercarbia held, we would have needed a total of 129,874 patients in our cohort study to detect a statistically significant difference. If instead, we had been able to include an equal number of patients with and without hypercarbia, we would have needed 19,625 patients in each group to detect a statistically significant difference in our observed effect size. However, it could be argued that our observed effect size is too small to be considered clinically meaningful. Therefore, in Table 2 we provide an exploration of potentially clinically meaningful effect sizes, and the corresponding sample sizes necessary for adequate power (80%) at an alpha of 0.05. If we assume that an equal number of patients with and without hypercarbia can be included in a future study, the number of patients needed in each group ranges from 17,413 to 602, depending on the effect size deemed clinically meaningful. For example, if a consensus could be made that in this setting a Relative Risk of 1.6 would be the smallest effect size of clinical importance, then a future study should include approximately 3,956 patients with hypercarbia and the same number without hypercarbia. This assumes that a simple relative risk would be the primary analysis, with no adjustment needed for potential confounding effects, such as differences in patient demographics or comorbidities.

**Table 2:** Sample sizes needed for potentially clinically meaningful effect sizes PJI: Prosthetic joint infection

Percent of patients without hypercarbia who develop PJI	Percent of patients with hypercarbia who develop PJI	Corresponding Relative Risk	Number of patients needed in each exposure group
1.6%	2.0%	1.26	17,413
1.6%	2.5%	1.58	3,956
1.6%	3%	1.90	1,859
1.6%	3.5%	2.23	1,136
1.6%	4%	2.56	794
1.6%	4.5%	2.90	602

## Discussion

Within this study, 1.6% of lower extremity TJR procedures had a PJI or wound complication within one year postoperatively. Although this includes both PJI and wound infection, this still falls within the nationally reported rates for PJI of 0.3-2% [7,8].

To our knowledge, this is the first study examining the relationship between bicarbonate levels and PJI in TJR patients. A previous study explored transcutaneous partial pressure of oxygen (TcPO_2_) at the total knee and hip replacement incision sites and found that TcPO_2_ significantly decreased from before surgery to postoperative day two, then returned to baseline after two months [9]. Though that study did not explore infection rates, low TcPO_2_ has been associated with an increased risk of healing complications in lower limb amputations [10]. In our study, hypercarbia was hypothesized to be a possible surrogate for poor tissue oxygenation. However, we were unable to demonstrate a correlation between hypercarbia and wound complications or PJI.

Our study was consistent with previous studies demonstrating that HbA1c greater than 7 and smoking may increase the risk of PJI. A study using the Danish Hip Arthroplasty Registry found a significantly higher risk of total hip revision due to deep infection in diabetic patients, especially type 2 diabetics, when compared to non-diabetics [11]. Additionally, in a previous meta-analysis, the risk of PJI after primary total hip and total knee arthroplasty was found to be significantly higher for both current and former tobacco users, with current smokers having the highest risk [12]. However, none of these studies explored hypercarbia or TcPO_2_ as potential risk factors or confounders for PJI.

Although we did not observe a correlation between hypercarbia and wound complications or PJI, several conditions that are known to result in hypercarbia have been identified as risk factors for poor wound healing. One study of total hip replacements found that chronic obstructive pulmonary disease (COPD) increased 90- day mortality, mortality at one year, infections within the first year post-operatively, and 30-day readmissions [13]. Asthma is another common chronic respiratory comorbidity that may lead to alveolar hypoventilation and hypercapnia, potentially resulting in poor lung, skeletal muscle, and innate immune system function [14]. Furthermore, chronic steroid use, which is often used to treat severe asthma, has been shown to impair wound healing and could potentially increase wound complications [15]. Both obstructive sleep apnea (OSA) and obesity have been found to result in hypercarbia [16,17]. OSA has been shown to increase the length of hospital stay, medical complications including surgical site infections, and healthcare costs for patients with total hip replacements after femoral neck fracture [18]. Multiple studies have demonstrated that obesity increases the risk of PJI in TJR [19,20]. While this evidence intuitively suggests that poor oxygenation is associated with an increased risk of infection, the exact mechanism remains elusive.

There are several limitations of our present study. First, all data were collected retrospectively from electronic medical records. Therefore, there is an inherent risk of missing or inaccurate data as well as a risk of selection bias. Secondly, there is a multitude of reasons for both acute and chronic hypercarbia. For this specific study, our intent was first to investigate whether there was a correlation between hypercarbia and PJI and wound complications. Future research of a much larger cohort is needed to determine if specific causes or conditions that lead to hypercarbia represent risk factors for PJI. Finally, our primary outcome of PJI and our primary exposure to hypercarbia are both rare events. In our study sample of 1,690, only 27 patients had a PJI or wound complication, and only 155 patients experienced hypercarbia. A post-hoc power analysis showed that our study would have needed a much larger and arguably unrealistic sample size to detect a statistically significant difference at our observed effect size. However, we would argue that our observed effect size was too small to be considered clinically important. To address this, we report the sample sizes required to detect potentially clinically meaningful effect sizes in Table 2.

## Conclusions

Although it was postulated that preoperative hypercarbia may indicate poor oxygenation and, therefore, correlate with increased risk of PJI or wound complications, this was not demonstrated in our study. The results of this study provide preliminary evidence that preoperative hypercarbia may not be correlated with an increase in the risk of PJI or wound complications. However, future research should involve larger institutions, multiple institutions, or comprehensive population-based databases in order to increase sample sizes and ensure adequate power to detect a clinically meaningful effect size. Future research should also explore other potential measures of tissue oxygenation and capacity to heal, such as transcutaneous partial pressure of oxygen or SPY technology (Novadaq Technologies Inc, Bonita Springs, Fla), as well as specific medical comorbidities that may affect the serum bicarbonate levels.
